# Inter-site and interpersonal diversity of salivary and tongue microbiomes, and the effect of oral care tablets

**DOI:** 10.12688/f1000research.27502.2

**Published:** 2021-04-09

**Authors:** Hugo Maruyama, Ayako Masago, Takayuki Nambu, Chiho Mashimo, Kazuya Takahashi, Toshinori Okinaga

**Affiliations:** 1 Department of Bacteriology, Osaka Dental University, Hirakata, Osaka, 573-1121, Japan; 2 Department of Geriatric Dentistry, Osaka Dental University, Hirakata, Osaka, 573-1121, Japan

**Keywords:** oral care tablet, oral microbiome, actinidin, QIIME 2, amplicon sequence variants (ASVs)

## Abstract

**Background: **Oral microbiota has been linked to both health and diseases. Specifically, tongue-coating microbiota has been implicated in aspiration pneumonia and halitosis. Approaches altering one's oral microbiota have the potential to improve oral health and prevent diseases.

**Methods:** Here, we designed a study that allows simultaneous monitoring of the salivary and tongue microbiomes during an intervention on the oral microbiota. We applied this study design to evaluate the effect of single-day use of oral care tablets on the oral microbiome of 10 healthy individuals. Tablets with or without actinidin, a protease that reduces biofilm formation *in vitro*, were tested.

**Results:** Alpha diversity of the tongue microbiome was significantly lower than that of the salivary microbiome, using both the number of observed amplicon sequence variants (254 ± 53 in saliva and 175 ± 37 in tongue; *P* = 8.9e-7, Kruskal–Wallis test) and Shannon index (6.0 ± 0.4 in saliva and 5.4 ± 0.3 in tongue; *P* = 2.0e-7, Kruskal–Wallis test). *Fusobacterium periodonticum*, *Saccharibacteria sp. 352*, *Streptococcus oralis *subsp *. dentisani*, *Prevotella melaninogenica*, *Granulicatella adiacens*, *Campylobacter concisus*, and *Haemophilus parainfluenzae* were the core operational taxonomic units (OTUs) common to both sites. The salivary and tongue microbiomes of one individual tended to be more similar to one another than to those of other individuals. The tablets did not affect the alpha or beta diversity of the oral microbiome, nor the abundance of specific bacterial species.

**Conclusions:** While the salivary and tongue microbiomes differed significantly in terms of bacterial composition, they showed inter- rather than intra-individual diversity. A one-day usage of oral care tablets did not alter the salivary or tongue microbiomes of healthy adults. Whether the use of oral tablets for a longer period on healthy people or people with greater tongue coating accumulation shifts their oral microbiome needs to be investigated.

## Introduction

Oral microbiota is a collection of microorganisms that reside in the oral cavity. It has been linked to the promotion of both health and diseases ^1, 2^. Among the different tissues in the oral cavity, the tongue is considered a dominant source of oral microbial populations ^3, 4^. Further, tongue coating is proposed to cause oral malodor ^5^ or, upon sudden dissociation, aspiration pneumonia in elderly people with impaired defense mechanisms ^6, 7^. In addition, the tongue coating is a risk indicator of aspiration pneumonia in edentate individuals ^8^.

A variety of methods to reduce tongue coating have been developed and tested to reduce oral malodor ^9, 10^. Mechanical removal of the tongue coating using tongue brushes or tongue cleaners is one such popular method ^9, 11^. Other methods include using antimicrobials, e.g., in gels or mouthwashes, or using oral tablets ^10, 12^.

The tongue microbiota in elderly individuals has been classified into several types with characteristic bacterial composition. These types correlate with the risk to aspiration pneumonia ^4, 13^. Therefore, methods that could alter the tongue microbiota to a healthy microbiota type could contribute to oral health. We have previously reported that tongue brushing does not alter the alpha or beta diversity of oral microbiota in healthy adults ^14, 15^. By contrast, according to a recent study, the use of oral care tablets decreases the amount of volatile sulfur compounds (VSCs) produced by bacteria ^16^. Further, oral care tablets that contain actinidin, a cysteine protease found in kiwifruit, reduce oral biofilm formation *in vitro* ^12^. However, it is not clear whether these interventions affect the oral microbiota as a whole or the abundance of specific bacteria.

In the current study, we examined the effect of oral care tablets with and without actinidin on the salivary and tongue microbiomes of healthy individuals. We also investigated the diversity of the salivary and tongue microbiomes, and interpersonal microbiome diversity. We show (1) that alpha diversity of the salivary microbiome was greater than that of the tongue microbiome, (2) that an individual’s salivary and tongue microbiomes were more similar to one another than to those of another individual, and (3) that the oral care tablets did not affect the oral microbiomes in the population tested. These findings add to the knowledge of the interpersonal diversity and dynamics of the oral microbiota in humans.

## Methods

Ten healthy adults participated in the study, with three different treatments tested: two different types of oral tablets (with or without protease), and a negative control (no tablet). For the tablet treatments, saliva and tongue coating were collected between October 2016 and November 2017 at participants’ home (mainly in Osaka, Japan, and in some cases, nearby prefectures). DNA extraction and data analysis were conducted at the Department of Bacteriology, Osaka Dental University (Hirakata, Japan).

### Participants

Participants were recruited from faculty members and graduate students working at the Osaka Dental University hospital, as well as from dentists who were acquainted with an author of this study. Ten healthy volunteers (6 males and 4 females; age: 27–60 years [39.8 ± 3.1 (mean ± SD)]) were enrolled in the study and were anonymized randomly as A–G, O, Q, and R ( Table 1). The inclusion criteria were as follows: healthy men and women over 20 years of age. The exclusion criteria were as follows: daily smoking, treatment with local or systemic antibiotics within 1 month prior to the study, and allergy to kiwifruit. The exclusion criteria of one month for antibiotic treatment was set based on previous reports on the robustness and resilience of salivary microbiome ^17, 18^. For example, change in microbiome caused by exposure to clindamycin lasted up to 1 month in saliva ^18^. According to the medical questionnaire, (1) none of the participants were undergoing or planning treatment for dental caries or periodontal disease, (2) there were no participants who were suffering from diabetes, chronic kidney disease, lung diseases, malignant tumors, etc., or who were visiting hospitals or taking medication, and (3) none of the participants experienced frequent thirst. The method and objective of this study were explained to the participants, who provided written informed consent before participating. The Osaka Dental University Medical Ethics Committee approved this study (approved on 3/31/2015; approval number 110864) and the investigations were conducted following the rules of the Declaration of Helsinki. The committee did not consider the study to be interventional in nature and therefore is not a clinical trial.

**Table 1.** Demographic data of the participants.

Participant	Age	Gender	Ethnicity
A	50	Female	Asian
B	45	Male	Asian
C	42	Male	Asian
D	35	Female	Asian
E	60	Female	Asian
F	28	Male	Asian
G	27	Male	Asian
O	27	Female	Asian
Q	39	Male	Asian
R	45	Male	Asian

### Oral tablets

Two types of oral care tablets for tongue cleaning were tested in the current study. One type contained actinidin, a cysteine protease extracted from kiwifruit (“protease tablet”) and the other did not (“plain [placebo] tablet”). Both tablets were provided by Ezaki Glico Co., Ltd (Osaka, Japan). The protease tablets were identical to those marketed as BREO EX (Ezaki Glico Co.). Tablet composition was described previously ^12^. To use the tablets, the participants placed one tablet on the dorsum of the tongue and waited until it dissolved naturally. One tablet takes approximately 5–7 min to completely dissolve.

### Study design

**Figure 1.**
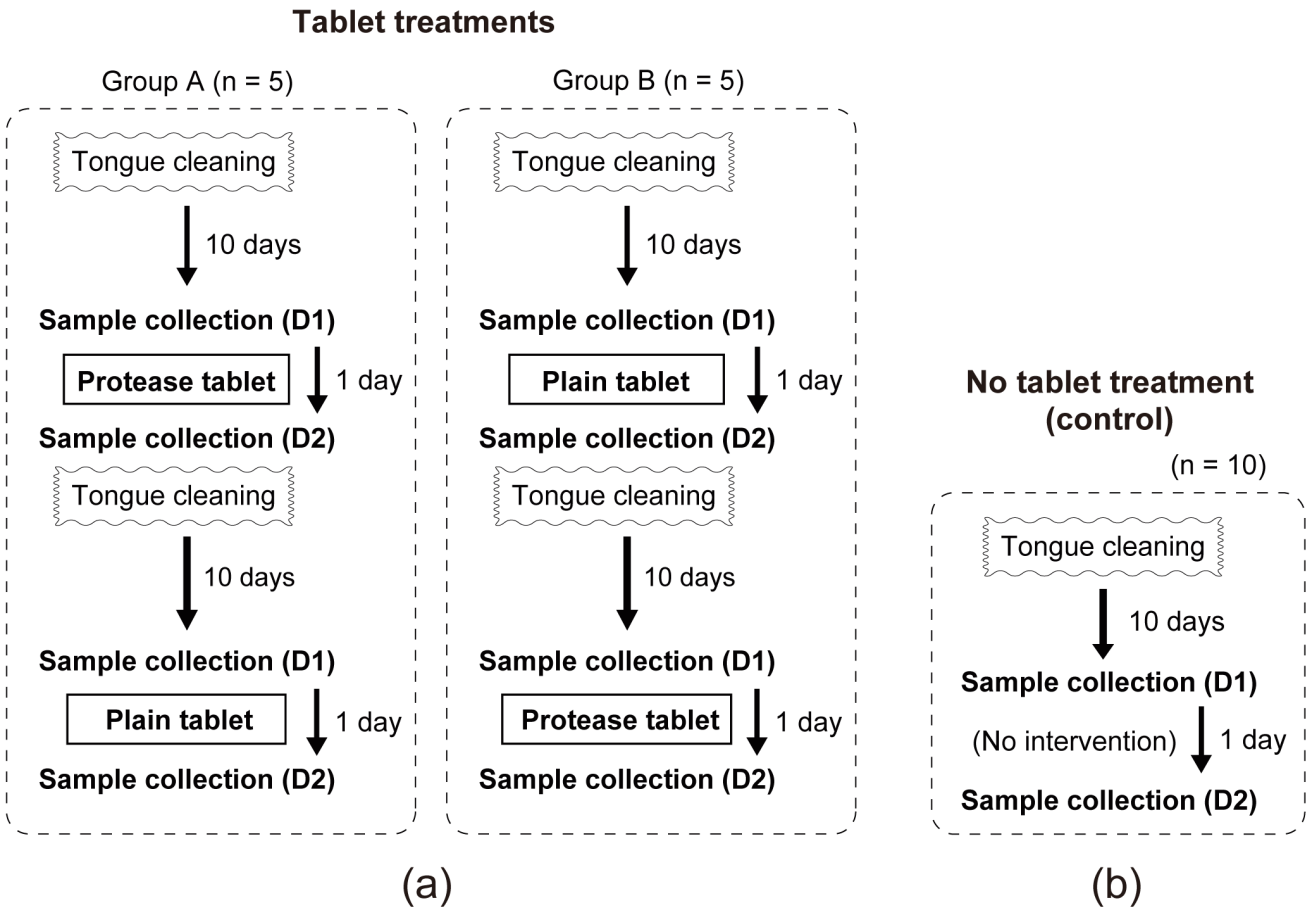
Schematic representation of the study design and trial schedule. ( **a**) Ten participants were randomly divided into two groups for the tongue tablet trials. ( **b**) Control (no tablet usage) treatment. The duration between the tablet treatments and the control experiment ranged from 10–60 days.

The study design is illustrated in Figure 1. The tongue tablet experiment was a placebo-controlled double-blind crossover study. The 10 participants were randomly divided into 2 groups of 5 participants each, by using computer-generated random numbers. All participants performed an initial tongue cleaning (by brushing) at the beginning of the study. The participants were asked not to eat, drink, or perform oral cleaning before each sampling. After a washout period of 10 days during which the participants did not perform any tongue cleaning, they collected their saliva and tongue coating into separate containers in the morning immediately after waking up (sample D1). Then, the participants in each group took tablets, with or without the protease. The participants and the researchers who analyzed the data were not informed about the tablet types given to the participants. The participants were asked to use the tablet three times on the day of the experiment—in the morning (between 9–12 am), in the afternoon (1–4 pm), and in the evening (7–10 pm)—taking one tablet each time. The following morning, the participants collected their saliva and tongue coating separately immediately after waking up (sample D2). After a washout period of 10 days, the participants took the other type of tablet that they had not previously received, and collected the saliva and tongue samples as before. Control experiments (no tablet usage) were conducted with the same participants, after they conducted treatment using the tablets. The duration between the tablet treatments and the control experiment ranged from 10–60 days, depending on the participant. In these experiments, after an initial tongue cleaning and a 10-day washout period, all participants collected samples on two consecutive days (D1 and D2) without taking any tablet in between.

### Sample collection

The saliva and tongue-coating samples were collected immediately after the participants woke up, in the morning of the day of tongue cleaning by tablet (D1) and the next morning (D2). The participants first collected 3 mL of saliva in a 25-mL sterile plastic tube. The tongue coating was collected by scrubbing the tongue with a swab and then soaking the tip of the swab in 0.6 mL of phosphate-buffered saline (PBS(-); Wako Pure Chemical Industries, Ltd., catalogue number 166-23555) to suspend the coating. Because this collection method involves scrubbing the tongue with a swab, the tongue coating was collected from the left half of the tongue for D1 and from the right half of the tongue for D2, to minimize the carryover effects of scrubbing. The collected samples were maintained at 4°C for up to 1 day and transported to the laboratory. The saliva samples (3 mL) were homogenized by repetitive pipetting. Then, 0.5-mL aliquots were transferred into sterile tubes. The saliva (0.5 mL) and tongue-coating (0.6 mL) samples were then centrifuged at 10,000 × *g* for 4 min. The supernatant was discarded and the pellet was stored at −20 °C until DNA extraction. All samples were frozen no later than on the day of D2 sampling.

### DNA extraction and library construction

Bacterial DNA was extracted from the pellets via chemical and mechanical lysis using a QIAamp UCP Pathogen Mini kit (QIAGEN, catalogue number 50214), as previously described ^19, 20^. Briefly, thawed pellets were immediately suspended in 0.5 mL of ATL buffer containing the optional DX reagent, transferred to a Pathogen Lysis Tube S, and then homogenized using a Mixer Mill MM 301 (Retsch) for 3 min at a vibrational frequency of 30 Hz. The manufacturer’s protocol was followed thereafter to complete the DNA purification. DNA was eluted in 50 μL of the AVE buffer (QIAGEN). DNA concentration was determined using a Quantus fluorometer (Promega) and a Qubit dsDNA BR Assay kit (Thermo Fisher Scientific, catalogue number Q32850). DNA was stored at –80 °C until use.

 Bacterial 16S ribosomal DNA amplification and library construction were performed according to the 16S Metagenomic Sequencing Library Preparation guide supplied by Illumina (part No. 15044223_B), as previously described ^19^. The V3–V4 region of the 16S ribosomal RNA gene was amplified by polymerase chain reaction (PCR) with a thermal cycler MJ-Mini (Bio-Rad Laboratories), using primers 341F (5’-TCGTCGGCAGCGTCAGATGTGTATAAGAGACAGCCTACGGGNGGCWGCAG-3’) and 806R (5’-GTCTCGTGGGCTCGGAGATGTGTATAAGAGACAGGGACTACHVGGGTWTCTAAT-3’) (custom-synthesized by Invitrogen), and Premix Ex Taq Hot Start Version (Takara Bio, catalogue number RR030A). The thermal cycling conditions were initial denaturation at 98 °C for 10 s, followed by 25 cycles at 98 °C for 10 s, 55 °C for 30 s, and 72 °C for 1 min (the first PCR step). The underlined nucleotides served as primer sequence parameters to extract the V3–V4 region for feature classifier training (see next section). The amplicons were purified using AMPure XP beads (Beckman Coulter, catalogue number A63880). Sequencing adapters containing 8-bp indices were incorporated at the 3’- and 5’-ends of the purified amplicons during a second PCR step. The amplicons were again purified using the AMPure XP beads, and then quantified using a Quantus fluorometer (Promega) and a Qubit dsDNA HS Assay kit (Life Technologies, catalogue number Q32851). After pooling equimolar amounts of the amplicons, 5% of an equimolar amount of PhiX DNA (PhiX Control v3, Illumina, catalogue number FC-110-3001), was added. The obtained library was pair-end sequenced at 2 × 250 bp using a MiSeq Reagent Kit v2 (Illumina, catalogue number MS-102-2001) and the Illumina MiSeq platform. Sequencing was performed over seven independent runs at the Oral Microbiome Center (Takamatsu, Japan), followed by demultiplexing. Raw nucleotide sequences are available at DDBJ/EMBL-EBI/NCBI database under the accession number DRA010849.

### Sequence processing and data analysis

Demultiplexed paired-end sequences were processed using QIIME 2 (v.2020.2) and its associated plugins ^21^ in a Docker container. Sequences obtained from independent Miseq runs were denoised separately using DADA2 (via q2-dada2) ^22^ applying previously-optimized parameters ^14^ (trim-left-f = 20; trim-left-r = 20; trunc-len-f and trunc-len-r were set between 241 and 248 depending on the sequence quality; other parameters followed the default settings, including chimera-method = “consensus”). The resulting exact amplicon sequence variants (ASVs) were merged (via q2-feature-table). For taxonomy assignment to each ASV, a naïve Bayes taxonomy classifier trained (via q2-feature-classifier) ^23^ on the V3–V4 region of the 16S rRNA sequences in the expanded human oral microbiome database ( eHOMD; v.15.2) ^24^ was used. All ASVs were aligned using MAFFT ^25^ and used to construct a phylogeny with FastTree 2 (via q2-phylogeny) ^26^. Sample metadata format was validated using the cloud-based tool Keemei ^27^.

Alpha diversity was assessed by calculating the number of observed features (ASVs) and the Shannon index (via q2-diversity), after samples were subsampled without replacement (rarefied) to 40,000 sequences per sample. The non-parametric Kruskal–Wallis test was used to test for significant differences in alpha diversity between sample groups ( *P* < 0.05).

Beta diversity was computed based on the unweighted UniFrac distance ^28^ (via q2-diversity) and visualized as three-dimensional principal coordinate analysis (PCoA) plots using EMPeror (via q2-emperor) ^29^. Permutational analysis of variance (PERMANOVA) ^30^ was used to test the significant difference in bacterial composition among samples ( *P* < 0.05). Differential abundance of bacterial taxonomic groups was tested using the analysis of composition of microbiomes (ANCOM) (via q2-composition) ^31^.

Clustered operational taxonomic unit (OTU) table was also used to calculate the beta diversity and differential abundance of specific taxonomic groups. Open-reference clustering was chosen for a high-quality taxonomic assignment to a curated database ^32^. Here, the entire ASVs were clustered into OTUs by open-reference picking (via q2-vsearch) ^32^ using the V3–V4 region of 16S rRNA sequences in the eHOMD v.15.2 as a reference, with 99% identity threshold. A phylogenetic tree was constructed as described above. The R package treeio (v.1.12.0) ^33^ was used to change the OTU names within the phylogenetic trees, as per the requirement for the Rhea package (the software disallows OTU names starting with a number). Multidimensional scaling (MDS) based on generalized UniFrac distance ^34^ was performed to examine the difference in microbial composition among samples, using Rhea pipeline (v.1.1.3) ^35^. For differential abundance analysis, “Serial group comparisons” in Rhea was performed (abundance_cutoff = 0.2; prevalence_cutoff = 0.3; max_median_cutoff = 1) with significance cutoff of *P* < 0.05 in Kruskal-Wallis test.

Box plots that show the alpha diversities were generated using R packages ggplot2 (v.3.3.2) and ggpubr (v.0.4.0), with data retrieved from QIIME 2 artifacts using qiime2R (v.0.99.31). Heatmaps and box plots that show the relative abundance of microbial taxonomies, and a Venn diagram that show core OTUs, were generated using ampvis2 (v.2.6.5) ^36^. R (v.4.0.2) and RStudio (v.1.3.959) were used for all analyses. Software, plugins, and R packages used in this study are listed in Table 2.

**Table 2.** List of software, plugin, R packages used in the study.

Name	Version	URL
QIIME 2	2020.2	https://qiime2.org/
Keemei		https://keemei.qiime2.org/
R	4.0.2	https://www.r-project.org/
Rstudio	1.3.959	https://rstudio.com/
Rhea	1.1.3	https://github.com/Lagkouvardos/Rhea
ampvis2	2.6.5	https://madsalbertsen.github.io/ampvis2/index.html
treeio	1.12.0	http://bioconductor.org/packages/release/bioc/html/treeio.html
qiime2R	0.99.31	https://github.com/jbisanz/qiime2R
ggplot2	3.3.2	https://ggplot2.tidyverse.org/
ggpubr	0.4.0	https://github.com/kassambara/ggpubr

QIIME 2 plugins are not listed here because they are associated with specific version of QIIME 2.

## Results

### Study overview

The study design is illustrated in Figure 1. Ten healthy adults participated in the study, with three different treatments tested: two different types of oral tablets (with or without protease), and a negative control (no tablet). For the tablet treatments, the saliva and tongue coating were collected before (D1) and after (D2) the intervention. Overall, 116 samples were collected (10 participants, three treatments, two sites [the saliva and tongue], and two sampling time points [D1 and D2], with four samples excluded because of insufficient amount of extracted DNA). The sample metadata are provided as underlying data (Table S1) ^37^.

DNA was extracted from each sample and the V3–V4 region of the 16S rRNA gene was PCR-amplified. The amplicons were paired-end sequenced using the Miseq platform. After quality control and error correction using DADA2, 11,260,102 reads corresponding to 5342 ASVs were obtained. Per-sample median was 89,923, with a maximum of 186,864 and a minimum of 46,422. Open-reference clustering, using the curated 16S rRNA sequences in the eHOMD v.15.2 database as the reference (at 99% identity threshold), grouped the sequences into 1210 OTUs. Either the full or clustered table was analyzed further, depending on the type of analysis performed, as described. The clustered OTU table is provided as underlying data (Table S2) ^38^.

### Inter-individual diversity of the salivary and tongue microbiomes

**Figure 2.**
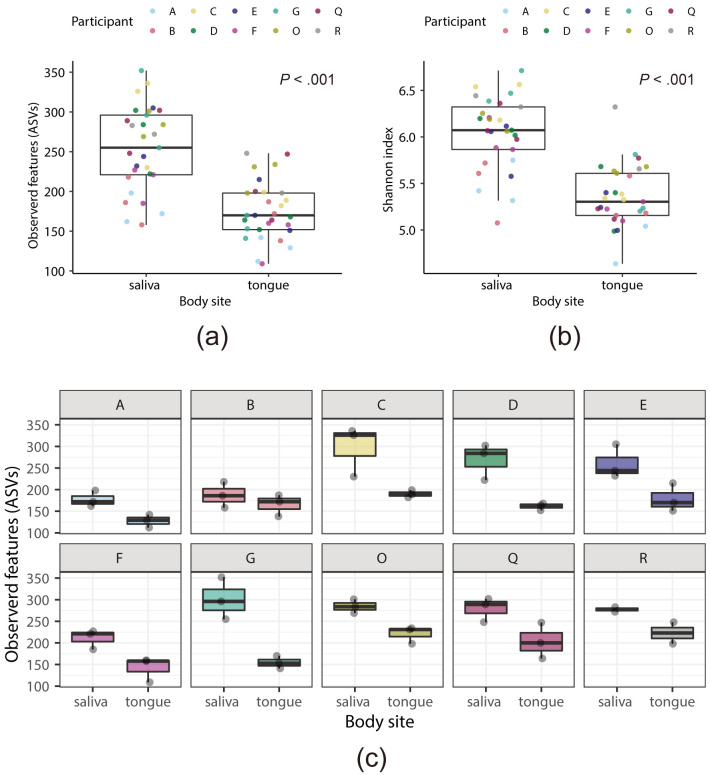
Alpha diversity of the salivary and tongue microbiomes. Data from the full amplicon sequence variants (ASV) table were used to calculate the alpha-diversity indexes. ( **a**, **b**) Thirty samples each from the salivary or tongue microbiome were compared (three D1 samples for each of 10 participants). The number of observed ASVs ( **a**) or Shannon index ( **b**) was used as the alpha-diversity measure. Each point indicates a sample. Colors of the points indicate different participants. ( **c**) Box plots of the number of observed ASVs in the salivary and tongue microbiomes (n = 3 each) for each participant.

We first analyzed the microbiome of the saliva and tongue coating, to determine the baseline for the study. In total, 30 D1 samples (10 participants, three independent treatments) of the saliva and tongue coating were analyzed. Alpha diversity of the tongue microbiome was significantly lower than that of the salivary microbiome, using both the number of observed ASVs (254 ± 53 in saliva and 175 ± 37 in tongue; *P* = 8.9e-7, Kruskal–Wallis test) ( Figure 2a) and Shannon index (6.0 ± 0.4 in saliva and 5.4 ± 0.3 in tongue; *P* = 2.0e-7, Kruskal–Wallis test) as measures ( Figure 2b). This was consistent with a previous report ^3^. The difference in diversity is probably associated with the tongue acting as a specialized niche for specific microorganisms, and the saliva containing a mixture of microbiota from different sites in the oral cavity. Interestingly, the number of observed ASVs varied among the individuals, ranging from approximately 170 to 325 in the saliva ( *P* = 0.027, Kruskal–Wallis test), and from approximately 125 to 225 in the tongue coating ( *P* = 0.022) ( Figure 2c), suggesting a difference in oral microbiome among individual.

**Figure 3.**
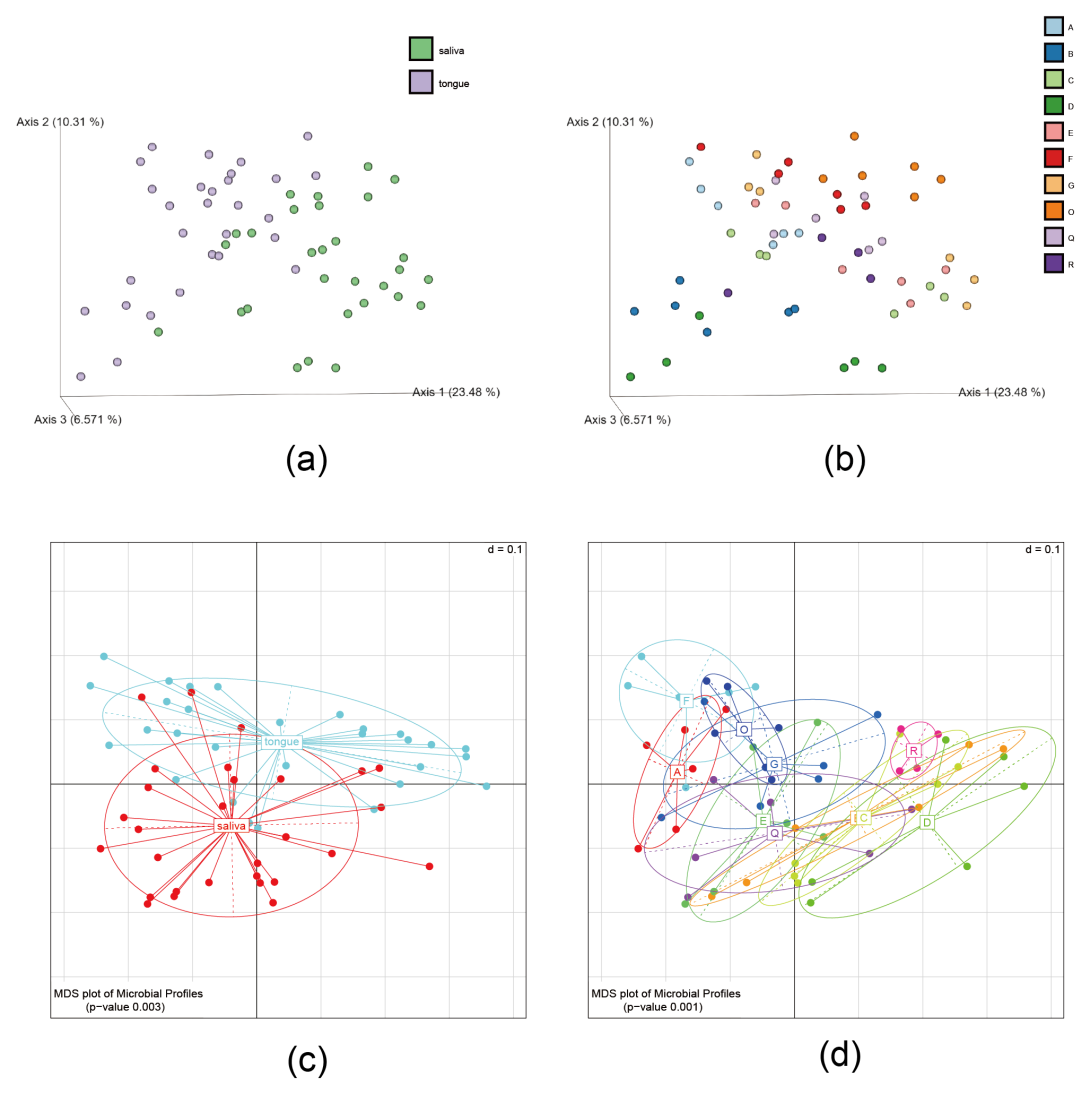
Beta diversity of the salivary and tongue microbiomes. ( **a**, **b**) Three-dimensional principle co-ordinate analysis (PCoA) plots were generated with EMPeror using the full amplicon sequence variants (ASV) table. While only samples corresponding to D1 are displayed (n = 58), the PCoA analysis included all (116) samples. ( **c**, **d**) multidimensional screening (MDS) plot of microbial profiles calculated based on generalized UniFrac distances using the clustered operational taxonomic unit (OTU) table. Each point indicates a sample. The points are colored according to the sampling site ( **a**, **c**) or the participant from whom the sample was obtained ( **b**, **d**).

We next assessed the differences in bacterial composition among samples (beta diversity) ( Figure 3). A significant difference between the salivary and tongue microbiomes was detected both in PCoA, based on unweighted UniFrac distances using the full ASV table ( *P* = 0.001, PERMANOVA) ( Figure 3a), and MDS, based on generalized UniFrac distances ^34^ using the clustered OTU table ( *P* = 0.003, PERMANOVA) ( Figure 3c).

The data also revealed a significant difference between individual microbiomes ( Figure 3b and 3d; *P* = 0.001, PERMANOVA using the clustered OTU table). As indicated by the plots in Figure 3, the similarity of the salivary and tongue microbiomes within an individual was greater than the similarity of the salivary or tongue microbiomes between individuals. This suggests there is stability in an individual’s oral microbiome, at least within the relatively short time period of the study (several weeks). This observation is consistent with earlier studies that highlight the stability of an individual’s oral microbiome ^39^.

### Differential abundance of bacterial taxonomic groups in the salivary and tongue microbiomes

**Figure 4.**
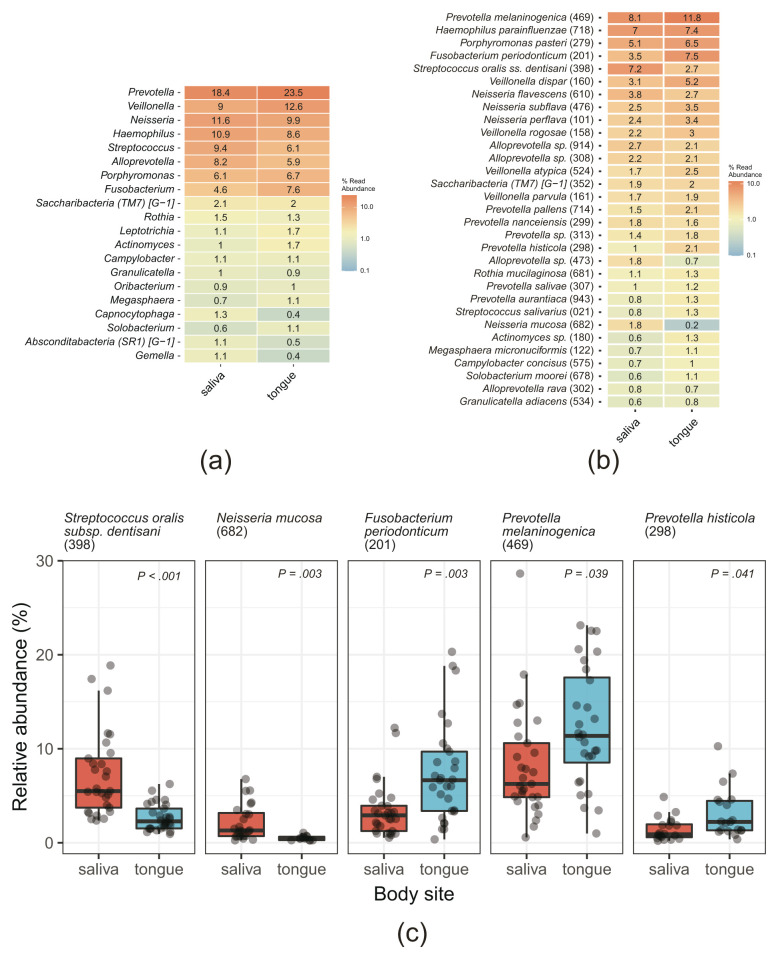
Abundances of bacterial taxa in the salivary and tongue microbiomes. The clustered operational taxonomic unit (OTU) table was used for calculations. ( **a**, **b**) Heatmaps of the abundance of bacterial taxa at the genus ( **a**) or species ( **b**) levels. The % read abundances are indicated, together with a color gradient. In ( **b**), the counts of clustered OTUs with the same Taxon ID were aggregated. ( **c**) Box plots summarizing the abundance of OTUs that were differentially abundant ( *P* < 0.05, Kruskal–Wallis test with Benjamini–Hochberg adjustment) in the saliva and tongue samples at the species level are shown. Each dot indicates a sample. Taxon IDs in eHOMD are indicated in the parentheses.

The abundances of bacterial taxonomic groups at the genus or species levels in the salivary and tongue microbiomes, determined by the analysis of D1 samples from the three treatments and based on the clustered OTU table are summarized in Figure 4a and 4b. The eight most abundant genera were common to the salivary and tongue microbiomes, accounting for nearly 80% of both microbiomes (78.2% in the saliva and 80.9% in the tongue). These genera were *Prevotella* (18.4% and 23.5%, respectively), *Veillonella* (9.0% and 12.6%, respectively), *Neisseria* (11.6% and 9.9%, respectively), *Haemophilus* (10.9% and 8.6%, respectively), *Streptococcus* (9.4% and 6.1%, respectively), *Alloprevotella* (8.2% and 5.9%, respectively), *Porphyromonas* (6.1% and 6.7%, respectively), and *Fusobacterium* (4.6% and 7.6%, respectively) ( Figure 4a). The abundance of bacterial taxa at species level is shown in Figure 4b. *Prevotella melaninogenica* HMT-469 was most abundant in both microbiomes (8.1% in the saliva and 11.8% in the tongue), followed by *Streptococcus oralis* subsp. *dentisani* HMT-398 (7.2%) and *Haemophilus parainfluenzae* HMT-718 (7.0%) in the saliva, and by *Fusobacterium periodonticum* HMT-201 (7.4%) and *H. parainfluenzae* HMT-718 (7.4%) in the tongue ( Figure 4b).

Five OTUs were differentially abundant in the salivary and tongue microbiomes ( *P* < 0.05, Kruskal–Wallis test) ( Figure 4c). Among them, *S. oralis* subsp. *dentisani* HMT-398 (7.2% in the saliva and 2.7% in the tongue) and *Neisseria mucosa* HMT-682 (1.8% and 0.2%, respectively) were more abundant in the saliva, whereas *F. periodonticum* HMT-201 (3.5% and 7.4%, respectively), *P. melaninogenica* HMT-469 (8.1% and 11.8%, respectively), and *Prevotella histicola* HMT-298 (1% and 2.1%, respectively) were more abundant in the tongue ( Figure 4b and 4c).

### Core OTUs in the Salivary and Tongue Microbiomes

**Figure 5.**
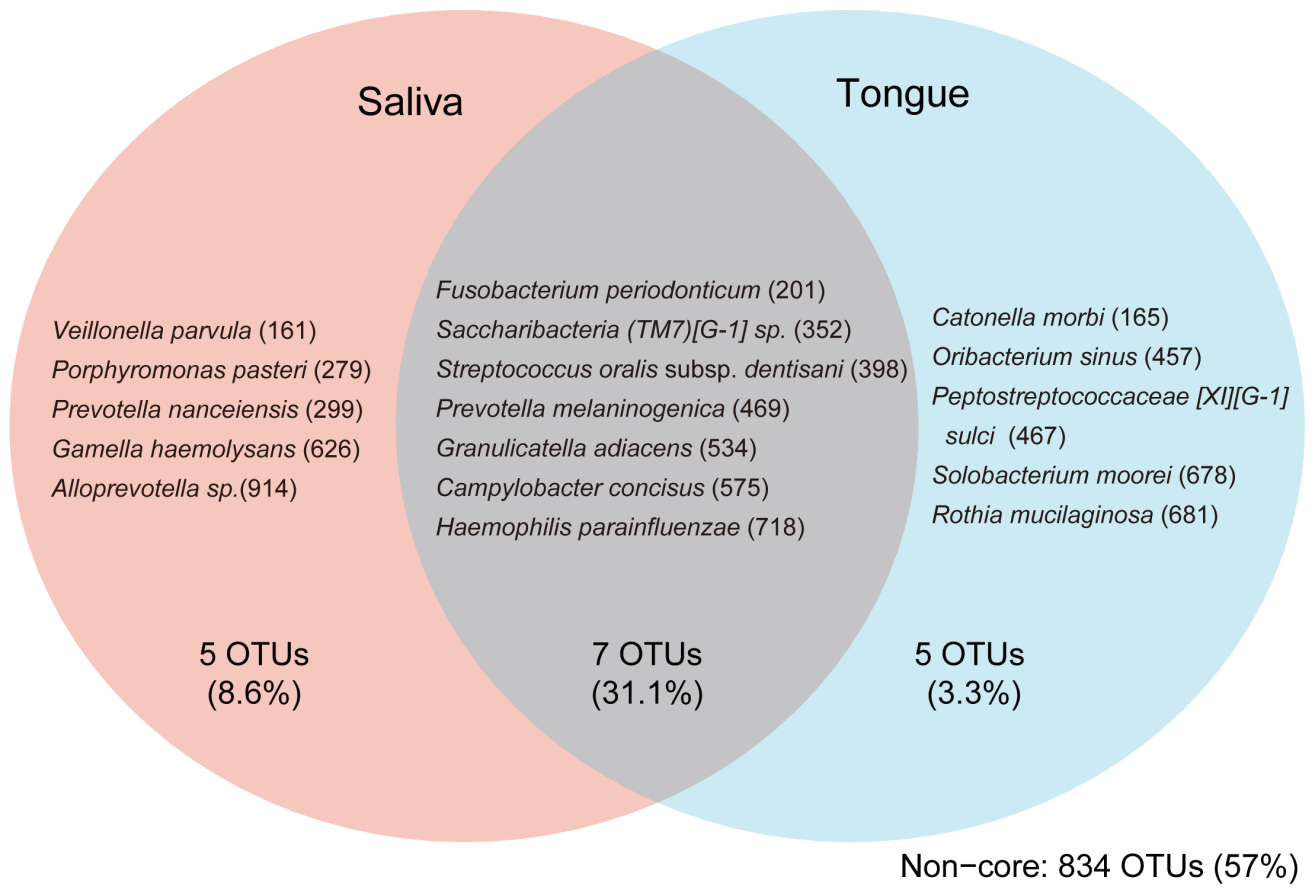
Venn diagram of the core oral operational taxonomic units (OTUs). OTUs present in ≥95% of samples (D1 samples, n = 58) in the indicated subset of oral sites are shown together with the average total abundance of the OTUs in the group. For example, *Veillonella parvula* was found in ≥95% of saliva samples and <95% of tongue samples. Taxon IDs in eHOMD are indicated in the parentheses.

To identify the core members of the oral microbiome, we focused on OTUs that were present in ≥95% of the saliva or tongue D1 samples. Seven OTUs were present in ≥95% of both, the saliva and tongue samples. These were *F. periodonticum* HMT-201, *Saccharibacteria (TM7) [G-1]* sp. HMT-352, *S. oralis* subsp. *dentisani* HMT-398, *P. melaninogenica* HMT-469, *Granulicatella adiacens* HMT-534, *Campylobacter concisus* HMT-575, and *H. parainfluenzae* HMT-718 ( Figure 5).

Further, we identified site-specific core OTUs, detected in ≥95% samples from one site but not from the other. The saliva-specific core OTUs were *Veillonella parvula* HMT-161, *Porphyromonas pasteri* HMT-279, *Prevotella nanceiensis* HMT-299, *Gemella haemolysans* HMT-626, and *Alloprevotella* sp. ** HMT-914. The tongue-specific core OTUs were *Catonella morbi* HMT-165, *Oribacterium sinus* HMT-457, *Peptostreptococcaceae [XI][G-1] sulci* HMT-467, *Solobacterium moorei* HMT-678, and *Rothia mucilaginosa* HMT-681 ( Figure 5).

### Effect of tablet taking on the salivary and tongue microbiomes

**Figure 6.**
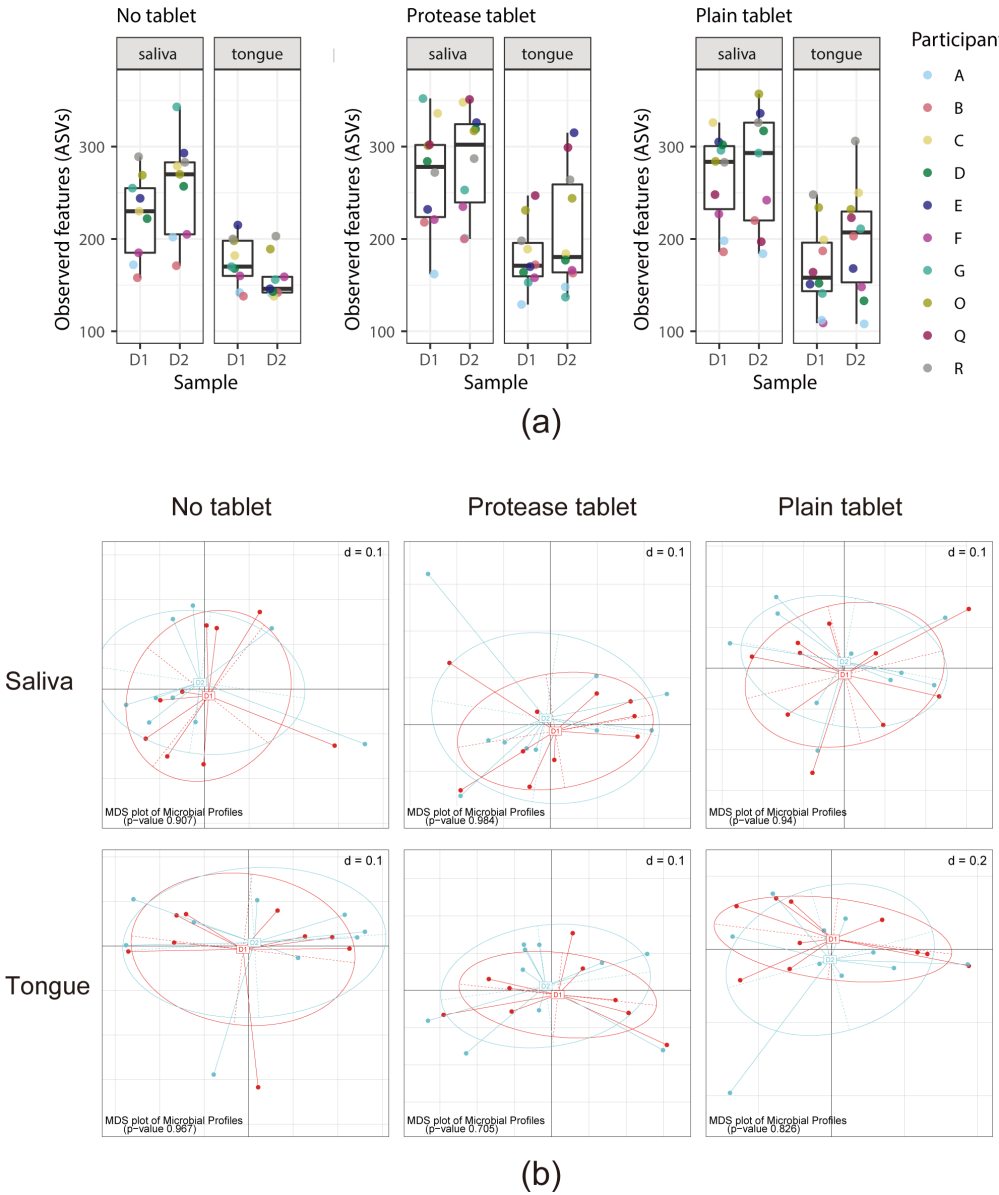
Effect of tablets on alpha and beta diversities of the salivary and tongue microbiomes. ( **a**) Box plots of the number of observed amplicon sequence variants (ASV) in the saliva and tongue D1 and D2 samples in the no tablet (left), protease tablet (middle), and plain tablet (right) treatments. Each point indicates a sample. ( **b**) multidimensional screening (MDS) analysis of beta diversity in D1 and D2 samples in the saliva (top panels) and tongue (bottom panels) in no tablet (left), protease tablet (middle), and plain tablet (right) treatments.

Using the above data as the base line, we finally assessed the effect of taking oral tablets (with or without protease) on the salivary and tongue microbiomes. Alpha diversity in D1 and D2 samples was not significantly different between any treatments ( Figure 6a). In the control (no tablet) treatment, whereas the observed number of ASVs seemed to slightly increase in the saliva and to slightly decrease in the tongue, they were not statistically significant ( Figure 6a). This indicates that some fluctuation of the oral microbiome may occur naturally. Further, MDS analysis indicated that the beta diversity between D1 and D2 samples was not significantly different in any treatment, for either the salivary or tongue microbiome ( *P* > 0.7, Kruskal–Wallis test) ( Figure 6B).

**Figure 7.**
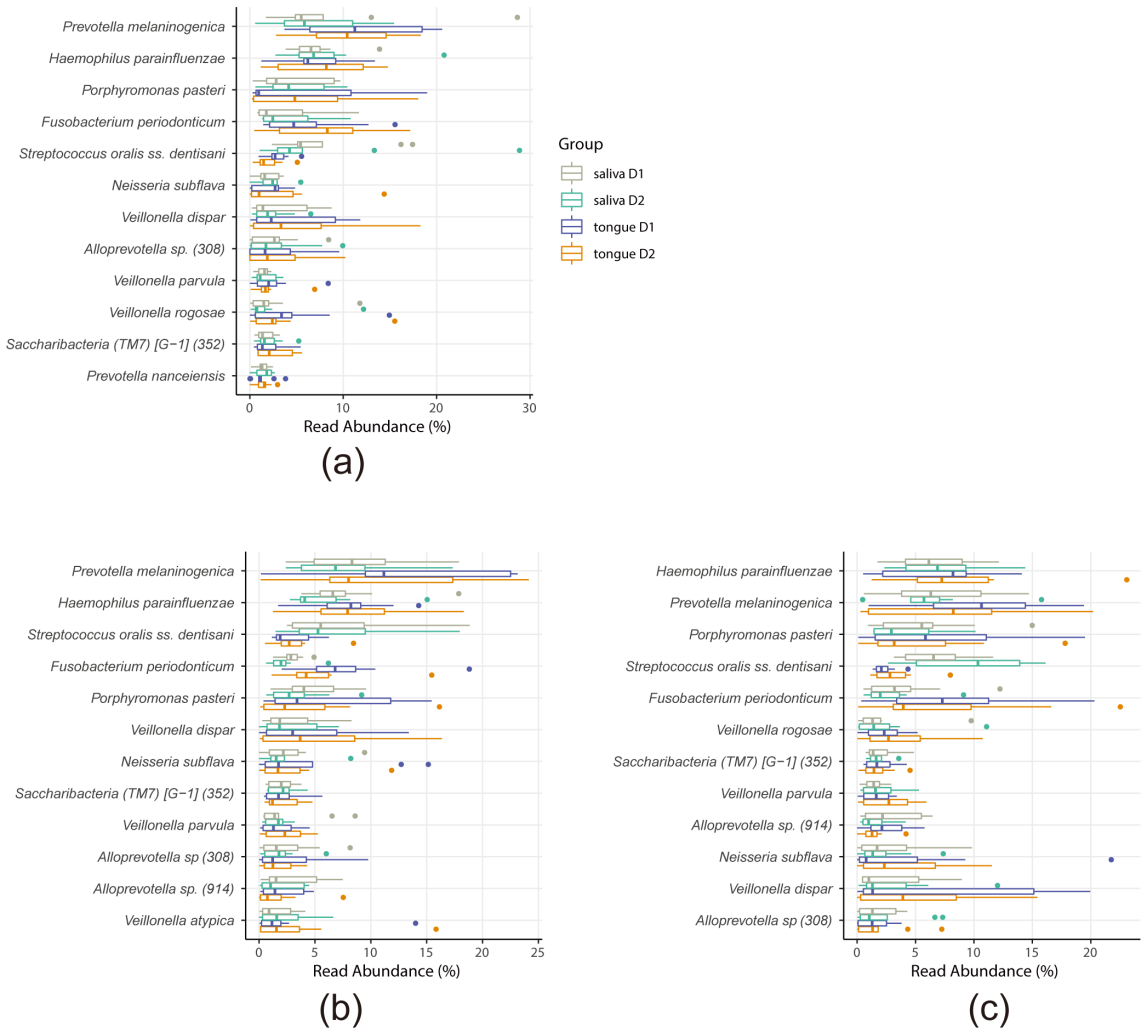
Box plots of OTU abundance. The abundances of top 12 operational taxonomic units (OTUs) based on the clustered OTU table for the no tablet ( **a**), protease tablet ( **b**), and plain tablet ( **c**) treatments are shown. The data are colored depending on the group (saliva or tongue, and D1 or D2). No bacterial species showed differential abundance before (D1) or after (D2). Taxon IDs in eHOMD are indicated in the parentheses.

We next examined whether any bacterial species were specifically impacted by oral tablet usage. Both ANCOM using the full ASV table or Kruskal–Wallis test using the clustered OTU table revealed that no OTU was differentially abundant before (D1) or after (D2) tablet use, in any of the treatments (no tablet, protease tablet, and plain tablet). The OTU abundance in each treatment group is summarized in Figure 7a–c. Although according to a recent study oral tablet use decreases the abundance of *Fusobacterium nucleatum* on the tongue of healthy young adults ^16^, we did not detect any significant decrease of OTUs that correspond to *F. nucleatum*. Further, in the current study, whereas 7.6% of all OTUs from the tongue microbiome were assigned to the genus *Fusobacterium* ( Figure 4a), the majority of them were classified as *F. periodonticum* (7.5% of total) and only <0.1% of all OTUs was assigned to *F. nucleatum* at species level.

## Discussion

The oral microbiota has been associated with specific diseases in susceptible populations. In the current study, we examined the effect of oral care tablet use, with or without actinidin, on the salivary and tongue microbiomes. We showed that whereas there are some differences between the tongue and salivary microbiomes, the microbiomes were not affected by the oral tablet use, regardless of the tablet type. This does not preclude the possibility that a persistent oral tablet use would alter the oral microbiome. Controlled alteration of the oral microbiome has potential for disease prevention.

We here identify the core OTUs that are common between saliva and tongue ( Figure 5). Among these OTUs, *S. oralis* and *Campylobacter* sp. have been previously determined to be the core OTUs common in the saliva and tongue ^3^. *F. periodonticum* and *Granulicatella adiacens* have been found in tongue microbiome of adults, and *P. melaninogenica* and *H. parainfluenzae* have been found in both the infant and adult tongue ^40^. TM7 species have been identified in oral microbiomes including tongue ^41, 42^ and supragingival plaque ^43^. It should be noted that although the high prevalence of *Saccharibacteria (TM7) [G-1]* sp. HMT-352 (≥95% in both saliva and tongue), as shown in our present study, has not been reported previously, it could also be a result of clustering similar sequences into a single OTU. Although there are some differences in the classification of the core or predominant oral OTUs between the current and other studies ^1, 3^, the majority of the species were identified as the core oral OTUs across the studies. Since low-abundance rather than highly abundant OTUs may contribute more to the difference in oral bacterial communities ^44, 45^, detailed analysis of low-abundance OTUs would be important in future research. *O. sinus* and *S. moorei* were previously classified as core OTUs common to the salivary and tongue microbiomes ^3^, but in our present study were identified as tongue-specific. This seems reasonable considering that *S. moorei* plays an important role in halitosis ^46– 48^. The effective separation of saliva- and tongue-specific OTUs suggest the usefulness of our study design in analyzing the salivary and tongue microbiomes simultaneously.

Our present study shows a variety among individuals in the number of observed ASVs in the salivary and tongue microbiomes. On the contrary, a significant interpersonal diversity in the supragingival plaque and salivary microbiomes, but not in the tongue plaque microbiomes was previously reported, using Faith’s phylogenetic diversity as a measure ^3^. Since the same V3–V4 region was targeted for amplicon sequencing in both studies, the discrepancy concerning the interpersonal differences in tongue microbiome might be associated with the differences in the alpha-diversity measure used, and/or in the participants’ age (25.3 ± 3.1 years in Hall *et al.* study ^3^ and 39.8 ± 10.9 years [mean ± SD] in the current study [see Methods]).

The tongue microbe is associated with various diseases, including halitosis ^49, 50^ and aspiration pneumonia ^4^; alterations in salivary microbiome has also linked to increasing numbers of oral and non-oral diseases ^51^. With the advancement of microbiome studies, methods to predict host traits that predispose to various diseases or conditions based on microbiome analysis have been developed ^52, 53^. Lu *et al.* have shown that tongue coating microbiome data can be used to distinguish individuals with pancreatic head carcinoma (PHC, one of pancreatic adenocarcinoma which occurs in the head of the pancreas) from healthy subjects ^41^. Although the evaluation of an individual’s disease status based on the tongue microbiota data is possible, the collection methods of the tongue coating samples may not be reliable when performed by a non-specialist, especially because of the anterior to posterior gradient of the bacterial communities in the tongue surface ^45^. We here showed that the microbiomes of the saliva and tongue of an individual tend to be more similar to one another than to the salivary or tongue microbiomes from other individuals. Considering this fact together with the stability of oral microbiome over a prolonged period of time ^39^, salivary collection could perhaps be used in the future as a standard method to predict diseases associated with the tongue coating microbiota, as well as those linked to that of the saliva.

Oral care tablets have been previously shown to reduce tongue coating load and VSCs ^12, 16^. Here we analyzed the effect of oral care tablets on the salivary and tongue microbiomes. To avoid individual varieties in the amount of tongue coating or saliva flow affecting the analysis, we recruited only healthy adults to participate in this study. We did not detect any significant differences in the alpha diversity, beta diversity, or abundance of specific OTUs at species level after oral tablet use. There are several possible explanations for these observations. First, the participants of the current study were healthy adults with no apparent tongue coating accumulation. Although accumulated tongue coating could be reduced with oral tablets ^12^, the amount of tongue coating analyzed herein may have been insufficient for detecting the differences in the microbiota. Second, the tablet intervention period in the current study was only 1 day and the samples were collected 1 day after the tablet use. In contrast, twice daily tongue scraping for three days, together with sampling within 15 mins after intervention, have shown to reduce the gram-negative anaerobes on the tongue ^9^. Although we chose to collect samples 1 day after the intervention, the 1-day period could have been long enough for the resilience of the oral microbiota to revert any shift in the oral microbiomes caused by the tablet use. Considering these factors, analyzing oral care tablet intervention in individuals with a higher tongue coating index and/or over a longer period of time together with immediate sampling after intervention may provide more information on whether and how oral care tablets alter the oral microbiota, contributing to the maintenance of oral health.

The impact of external agents on microbiome depends on the location of microbiome. For example, salivary microbiome is highly resilient against external agents including antimicrobials, compared to feces microbiome that is more easily affected ^18^. Thus, methods that can alter oral microbiome has been anticipated. Oral care tablets containing actinidin reduces tongue coating, and actinidin prevents biofilm formation by degrading cell-surface proteins *in vitro* ^12^. We here attempted to elucidate the effect of the protease, supplied in oral tablets, on the oral microbiome. Unfortunately, we were unable to assess the effect of the protease, because oral tablet treatments failed to alter the oral microbiome or specific bacterial taxa, regardless of the presence or absence of actinidin. As above, including participants with a higher tongue coating and a longer intervention period with immediate sampling may have allowed detection of the effect of actinidin in the tablets. Alternatively, an *in vitro* culturing system could be used to analyze the effect of actinidin on the oral microbiome, with the effects of the compound tested in a controlled manner. For example, nitric oxide ^19^ or statins ^54^ have been shown to alter the abundance of specific bacterial species. Using such system would allow the analysis of the effect of actinidin on oral microbiota separately from the effect of mechanical removal of the tongue coating.

Various lines of evidence suggest a link between oral microbiota and health or diseases ^1, 2, 55^. The current and other ^3^ studies have highlighted interpersonal differences in the oral microbiota. Several types of tongue microbiota have been shown to exist in individuals with different susceptibility to pneumonia ^4^. Hence, personalized treatment based on an individual’s oral microbiota is required, as has been already pointed out in the context of periodontal disease ^56^. Analysis of how different types of oral microbiota are affected by certain interventions (e.g., oral care tablet or antibiotic treatment) would enable a more precise control over the oral microbiome in the future. *In vitro* culturing systems mentioned above are powerful tools for elucidating responses of bacterial communities taken from different individuals to various interventions, and the contributing factors.

In conclusion, we have shown that while the salivary and tongue microbiomes differ significantly in terms of bacterial composition, they show inter- rather than intra-individual diversity, although it should be noted that the study has a limitation in the sample size of ten individuals. We have also identified bacterial species that are common to the salivary and tongue microbiome, as well as those that are specific to either of these. In addition, we showed that oral care tablets may not alter the bacterial composition of the saliva or the tongue, at least over short periods of time in healthy individuals. Considering the link between oral microbiota and health or disease, analyzing the differences in how individual oral microbiota responds to external factors will pave the way to more effective therapeutic and diagnostic approaches and, ultimately, contribute to the development of personalized dental medicine.

## Data availability

### Underlying data

Raw nucleotide sequences are available at DDBJ/EMBL-EBI/NCBI database under the accession number DRA010849.

Figshare: Table_S1_sample-metadata.tsv for "Inter-site and interpersonal diversity of salivary and tongue microbiomes, and the effect of oral care tablets". https://doi.org/10.6084/m9.figshare.13289618.v1 ^37^

Table_S1_sample-metadata.tsv: Columns indicate sample ID, participant, sampling date (D1 or D2), treatment, Miseq run number, and sampling body site (saliva or tongue) of each sample. The “treatment” column indicates, no tablet (E2), protease tablet (E3), or plain tablet (E4) treatments.

Figshare: Table_S2_clustered-OTU-table.tsv for "Inter-site and interpersonal diversity of salivary and tongue microbiomes, and the effect of oral care tablets". https://doi.org/10.6084/m9.figshare.13291535.v1 ^38^

Table_S2_clustered-OTU-table. After open-reference clustering, OTU table was constructed from the BIOM file using QIIME 2. Number of reads for each OTU in each sample are indicated, together with the bacterial taxonomy assigned to each OTU. OTU IDs are identical to matching HOMD Refseq IDs, except for those which did not match the database.

Data are available under the terms of the Creative Commons Zero "No rights reserved" data waiver (CC0 1.0 Public domain dedication).
