# Supplementary material for: Inter-site and interpersonal diversity of salivary and tongue microbiomes, and the effect of oral care tablets
Source: F1000Res. 2021 Apr 9;9:1477. Originally published 2020 Dec 17. [Version 2] doi: 10.12688/f1000research.27502.2 (PMC7921892; doi:10.12688/f1000research.27502.2)
Supplement: Peer review history

# Peer review: Reviewer response for version 2

**DOI:** 10.5256/f1000research.55793.r83021

**Published:** 2021-04-09

**Authors:** Takuichi Sato

**Affiliations:** 1 Division of Clinical Chemistry, Niigata University Graduate School of Health Sciences, Niigata, Japan

I do not have any new comments.

Is the work clearly and accurately presented and does it cite the current literature?

Yes

If applicable, is the statistical analysis and its interpretation appropriate?

I cannot comment. A qualified statistician is required.

Are all the source data underlying the results available to ensure full reproducibility?

Yes

Is the study design appropriate and is the work technically sound?

Yes

Are the conclusions drawn adequately supported by the results?

Yes

Are sufficient details of methods and analysis provided to allow replication by others?

Yes

Reviewer Expertise:

Oral Microbiology

I confirm that I have read this submission and believe that I have an appropriate level of expertise to confirm that it is of an acceptable scientific standard.

# Peer review: Reviewer response for version 1

**DOI:** 10.5256/f1000research.30395.r79136

**Published:** 2021-03-01

**Authors:** Priya Nimish Deo

**Affiliations:** 1 Department of Oral and Maxillofacial Pathology and Oral Microbiology, Bharati Vidyapeeth Deemed to be University Dental College and Hospital, Pune, India

It is a well designed study.

 Effect of one day use of oral care tablets on oral microbiome was studied. Monitoring of the salivary and the tongue microbiomes is conducted simultaneously during an interrvention. Their results showed that the alpha diversity was higher in the saliva than on the tongue without intervention. The tablets did not affect the diversity not the abundance of specific species. Studies need to be carried out for longer duration to study the shifts in the composition of the microbiome upon intervention.

Is the work clearly and accurately presented and does it cite the current literature?

Yes

If applicable, is the statistical analysis and its interpretation appropriate?

Yes

Are all the source data underlying the results available to ensure full reproducibility?

Yes

Is the study design appropriate and is the work technically sound?

Yes

Are the conclusions drawn adequately supported by the results?

Yes

Are sufficient details of methods and analysis provided to allow replication by others?

Yes

Reviewer Expertise:

Oral microbiome and oral cancer

I confirm that I have read this submission and believe that I have an appropriate level of expertise to confirm that it is of an acceptable scientific standard.

# Response

**Published:** 2021-04-02

**Authors:** Hugo Maruyama

**Affiliations:** Osaka Dental University, Japan

Thank you very much for your review and comment on our manuscript.

**Studies need to be carried out for longer duration to study the shifts in the composition of the microbiome upon intervention.**

 We agree that longer term study is needed to determine the impact of oral care tablets on the oral microbiome. This point is discussed in the "Discussion" section.

# Peer review: Reviewer response for version 1

**DOI:** 10.5256/f1000research.30395.r79337

**Published:** 2021-03-01

**Authors:** Rohit Kunnath Menon

**Affiliations:** 1 Division of Clinical Dentistry, School of Dentistry, International Medical University, Kuala Lumpur, Malaysia

Comment 1

 Abstract: The results section should provide values for the diversity and the names of at least the most important core OTUs.

Comment 2

 The authors may include a significant limitation of the study in the discussion section. Which is the sample size of ten individuals. The conclusions regarding the inter-participant differences rather than intra-individual variation is also previously well-established by studies with larger sample size. Hence it is ambitious to claim that this result is well-established with the small sample size.

Comment 3

 Another significant limitation is the the lack of clarity in how the individuals were deemed healthy orally as well as systemically. . The absence of clinical data on the caries and periodontal health status of each participant needs to be explained. Previous research clearly shows the impact of oral diseases in determining the microbiome of the oral cavity especially saliva.

Comment 4

 It is not surprising that one day treatment with the tablet did not significantly impact the temporal variation. Previous research has shown that even antibiotic treatment does not significantly impact salivary microbiome. The impact of the external agents on the microbiome should be discussed with inclusion of more of such previous investigations. The exclusion criteria of one month for antibiotic treatment should also be discussed with respect to previous available literature:

- R K Menon, A Gomez, B W Brandt, Y Y Leung, D Gopinath, R M Watt, W Crielaard, K E Nelson, M G Botelho. Long-term impact of oral surgery with or without amoxicillin on the oral microbiome-A prospective cohort study. Sci Rep. 2019 Dec 10;9(1):18761 ^1^
- Zaura, E. et al. Same Exposure but Two Radically Different Responses to Antibiotics: Resilience of the Salivary Microbiome versus Long-Term Microbial Shifts in Feces. mBio 6, e01693–01615 (2015) ^2^

Comment 5

 Please check the English grammar. For example: "We here analyzed the effect of oral care tablets on the salivary and tongue microbiome."

Is the work clearly and accurately presented and does it cite the current literature?

Yes

If applicable, is the statistical analysis and its interpretation appropriate?

Yes

Are all the source data underlying the results available to ensure full reproducibility?

Yes

Is the study design appropriate and is the work technically sound?

Yes

Are the conclusions drawn adequately supported by the results?

Yes

Are sufficient details of methods and analysis provided to allow replication by others?

Yes

Reviewer Expertise:

oral microbiome

I confirm that I have read this submission and believe that I have an appropriate level of expertise to confirm that it is of an acceptable scientific standard.

## References

[1] : Long-term impact of oral surgery with or without amoxicillin on the oral microbiome-A prospective cohort study. *Scientific Reports*.2019;9(1) : 10.1038/s41598-019-55056-3 10.1038/s41598-019-55056-3 31822712PMC6904678

[2] : Same Exposure but Two Radically Different Responses to Antibiotics: Resilience of the Salivary Microbiome versus Long-Term Microbial Shifts in Feces. *mBio*.2015;6(6) : 10.1128/mBio.01693-15 e01693-15 10.1128/mBio.01693-15 26556275PMC4659469

# Response

**Published:** 2021-04-02

**Authors:** Hugo Maruyama

**Affiliations:** Osaka Dental University, Japan

Thank you very much for your review and comments on our manuscript. Below is a point-by-point response to your comments.

**Comment 1**

**Abstract: The results section should provide values for the diversity and the names of at least the most important core OTUs.**

 We have included the values of alpha diversity and the names of the core OTUs in the abstract.

**Comment 2**

**The authors may include a significant limitation of the study in the discussion section. Which is the sample size of ten individuals. The conclusions regarding the inter-participant differences rather than intra-individual variation is also previously well-established by studies with larger sample size. Hence it is ambitious to claim that this result is well-established with the small sample size.**

 The following statement has been added to the last paragraph of the Discussion: ", although it should be noted that the study has a limitation in the sample size of ten individuals."

**Comment 3**

**Another significant limitation is the lack of clarity in how the individuals were deemed healthy orally as well as systemically. The absence of clinical data on the caries and periodontal health status of each participant needs to be explained. Previous research clearly shows the impact of oral diseases in determining the microbiome of the oral cavity especially saliva.**

 The following description has been added to the "Participants" section of the Methods: "According to the medical questionnaire, (1) none of the participants were undergoing or planning treatment for dental caries or periodontal disease, (2) there were no participants who were suffering from diabetes, chronic kidney disease, lung diseases, malignant tumors, etc., or who were visiting hospitals or taking medication, and (3) none of the participants experienced frequent thirst."

**Comment 4**

**It is not surprising that one day treatment with the tablet did not significantly impact the temporal variation. Previous research has shown that even antibiotic treatment does not significantly impact salivary microbiome. The impact of the external agents on the microbiome should be discussed with inclusion of more of such previous investigations. The exclusion criteria of one month for antibiotic treatment should also be discussed with respect to previous available literature:**

- R K Menon, A Gomez, B W Brandt, Y Y Leung, D Gopinath, R M Watt, W Crielaard, K E Nelson, M G Botelho. Long-term impact of oral surgery with or without amoxicillin on the oral microbiome-A prospective cohort study. Sci Rep. 2019 Dec 10;9(1):187611
- Zaura, E. et al. Same Exposure but Two Radically Different Responses to Antibiotics: Resilience of the Salivary Microbiome versus Long-Term Microbial Shifts in Feces. mBio 6, e01693–01615 (2015)2

The following description has been added to the "Participants" section of the Methods: "The exclusion criteria of one month for antibiotic treatment was set based on previous reports on the robustness and resilience of salivary microbiome [Zaura, E. et al. 2019;Menon RK et al. 2015]. For example, change in microbiome caused by exposure to clindamycin lasted up to 1 month in saliva [Zaura, E. et al.]."

 In addition, the following statement has been added to the sixth paragraph of the Discussion: "The impact of external agents on microbiome depends on the location of microbiome. For example, salivary microbiome is highly resilient against external agents including antimicrobials, compared to feces microbiome that is more easily affected [Zaura et al. mBio 6, e01693–01615 (2015)2]."

**Comment 5**

**Please check the English grammar. For example: "We here analyzed the effect of oral care tablets on the salivary and tongue microbiome."**

 The text has been changed to "Here, we analyzed the effect of oral care tablets on the salivary and tongue microbiomes." The entire manuscript has been checked by a native speaker.

# Peer review: Reviewer response for version 1

**DOI:** 10.5256/f1000research.30395.r76292

**Published:** 2020-12-22

**Authors:** Takuichi Sato

**Affiliations:** 1 Division of Clinical Chemistry, Niigata University Graduate School of Health Sciences, Niigata, Japan

Suggestions:

 Results

 Figure 4(b): The authors need to check the figure. There are THREE "Porphyromonas pasteri (279)" in the figure.

 Discussion

 Figure 5 should be cited in the second paragraph of the discussion.

 Typographical errors;

 Results

 P. 6: "Neisseria (11.6% and 9.9% respectively)"

 Insert a comma between "9.9%" and "respectively".

 P. 7: "F. periodonticum HMT-201 (3.5% and 7.5%, respectively."

 "7.5%" should read "7.4%".

 Figure 4(a): The authors need to check the figure. Absconditabacteria?

 References:

 The authors need to check the style of the references #2, 4, 7, 13, 17, 22, 26, 31, 37, 38, 41, 44, 50, 51 and 54.  (Do not use capital letters in the title of each reference.)

Is the work clearly and accurately presented and does it cite the current literature?

Yes

If applicable, is the statistical analysis and its interpretation appropriate?

I cannot comment. A qualified statistician is required.

Are all the source data underlying the results available to ensure full reproducibility?

Yes

Is the study design appropriate and is the work technically sound?

Yes

Are the conclusions drawn adequately supported by the results?

Yes

Are sufficient details of methods and analysis provided to allow replication by others?

Yes

Reviewer Expertise:

Oral Microbiology

I confirm that I have read this submission and believe that I have an appropriate level of expertise to confirm that it is of an acceptable scientific standard.

# Response

**Published:** 2021-04-02

**Authors:** Hugo Maruyama

**Affiliations:** Osaka Dental University, Japan

Thank you very much for your review and comments on our manuscript.

 Below is a point-by-point response to your comments.

**Suggestions:**

**Results**

**Figure 4(b): The authors need to check the figure. There are THREE "Porphyromonas pasteri (279)" in the figure.**

 Thank you for pointing this out. This was due to the multiple slightly different reference 16S rRNAs sequences present in the HOMD database used for open-reference clustering of ASVs into OTUs ( http://www.homd.org/index.php?name=HOMD&view=dynamic&oraltaxonid=279). Because the original figure was created based on counts per OTU, the table contained three " *Porphyromonas pasteri* (279)". In the revised figure, we aggregated the counts for OTUs with a common Taxon ID (in this case, HMT-279).

 An explanation was added to the figure legend: "In (b), count for clustered OTUs with common Taxon ID were aggregated."

**Discussion**

**Figure 5 should be cited in the second paragraph of the discussion.**

 Figure 5 is now cited as recommended.

**Typographical errors;**

**Results**

**P. 6: "Neisseria (11.6% and 9.9% respectively)"**

**Insert a comma between "9.9%" and "respectively".**

**P. 7: "F. periodonticum HMT-201 (3.5% and 7.5%, respectively."**

**"7.5%" should read "7.4%".**

 All typographical errors that were pointed out have been corrected.

**Figure 4(a): The authors need to check the figure. Absconditabacteria?**

 The problem of the left edge of the figure being cut off has been fixed.

**References:**

**The authors need to check the style of the references #2, 4, 7, 13, 17, 22, 26, 31, 37, 38, 41, 44, 50, 51 and 54. (Do not use capital letters in the title of each reference.)**

 The style of the references has been unified as pointed out.
